# Optimizing plant growth, nutrient uptake, and yield of onion through the application of phosphorus solubilizing bacteria and endophytic fungi

**DOI:** 10.3389/fmicb.2024.1442912

**Published:** 2024-07-25

**Authors:** Thangasamy Arunachalam, Komal Gade, Payal Arun Mahadule, P. S. Soumia, Venkadasamy Govindasamy, Suresh Janardhan Gawande, Vijay Mahajan

**Affiliations:** ^1^ICAR-Directorate of Onion and Garlic Research, Rajgurunagar, Pune, India; ^2^Division of Microbiology, ICAR-Indian Agricultural Research Institute, New Delhi, India

**Keywords:** onion yield, nutrient use efficiency, soil fertility, *Serendipita indica*, phosphate solubilizing bacteria, vesicular arbuscular mycorrhiza

## Abstract

**Introduction:**

The application of mineral fertilizers deteriorates soil properties and affects crop yield and nutritional properties. However, plant growth-promoting microorganisms (PGPM- *Serendipita indica*, phosphorus solubilizing bacteria (PSB), and vesicular arbuscular mycorrhizae (VAM)) have great potential to reduce fertilizers and improve soil fertility, crop yield, and nutrient uptake and mitigate the environmental effect of mineral fertilizers.

**Material and methods:**

Hence, a field experiment was conducted involving nine treatments to evaluate the effects of PGPM along with 50% or 100% of the recommended dose of fertilizers on plant growth, soil fertility, nutrient uptake, and onion productivity.

**Results and discussion:**

Results indicated that 100% RDF combined with *S. indica* or PSB led to improved plant growth, and higher nutrient concentrations in both leaves and bulbs of onions compared to RDF alone. Moreover, the application of 100% RDF with *S. indica* increased total dry matter yield by 11.5% and 7.6% in the 2018-2019 and 2019-2020 seasons, respectively, compared to 100% RDF alone. This treatment also resulted in the highest nutrient uptake, with N uptake increasing by 6.9%-29.9%, P by 13.7%-21.7%, K by 20.0%-23.7%, and S by 18.1%-23.4%. Additionally, the combination of 100% RDF with *S. indica* inoculation led to a notable increase in bulb yield, with increments of 16.2% and 13.9% observed in 2018-2019 and 2019-2020, respectively, compared to 100% RDF alone. Similarly, the application of 100% RDF along with PSB inoculation resulted in an increase in bulb yield by 7.2% and 9.4% in the respective years. However, VAM did not exhibit satisfactory performance or improvements in the onion crop.

**Conclusion:**

Overall, the study suggests that combining 100% RDF with *S. indica* or PSB can enhance onion productivity and nutrient use efficiency. The present study may open a new avenue of PGPM application in enhancing onion yield and improving the bulb quality as well as soil health. However, field trials across different regions and soil types are necessary to validate these findings for practical adoption by farmers.

## Introduction

Onion (*Allium cepa* L.) is the 4th economically most important vegetable crop grown worldwide (Torquato-Tavares et al., 2017). India is the leading producer of onions, producing 31.68 million tons from a cultivated area of 1.94 million hectares (FAO, 2024). Despite substantial production, the country faces challenges in onion productivity, with a low yield of 16.32 t ha^−1^ compared to the global average of 18.53 t ha^−1^. Among the many constraints for low productivity in onions, unbalanced nutrition is the main limiting factor. The indiscriminate use of fertilizers not only harms agricultural sustainability but also pollutes the environment (Jaiswal et al., 2022). Integrated Nutrient Management (INM) offers a comprehensive solution to address these imbalances, promoting soil health and maximizing crop yield by optimizing nutrient sources in an integrated approach (Qiu et al., 2023; Yang et al., 2024).

Historically, a blanket recommendation of 150:50:80:50 kg NPKS and 20 t farm yard manure (FYM) ha^−1^ was suggested to achieve a yield of 40–50 tons of onion bulbs per hectare. However, recent field experiments conducted at ICAR-DOGR demonstrated that applying 110:40:60:30 kg NPKS and 15 t FYM ha^−1^ produced yields comparable to the previous recommendation (Thangasamy and Lawande, 2015). This highlights the potential for improving nutrient management practices to enhance onion productivity.

Notably, nitrogen (N) applications, sourced from both organic and mineral fertilizers, typically range from 175–185 kg ha^−1^, whereas the crop’s actual requirement for optimal yield is around 90–100 kg N ha^−1^ to produce 40–50 tons of onion bulbs (Thangasamy, 2016). Nutrient uptake rates vary with growth stages; N demand is the highest during seedling production and the vegetative growth phase (Padhan et al., 2023; Abou Fayssal et al., 2024). Consequently, excess N applied can lead to leaching, denitrification, and increased vulnerability to pests and diseases (Sekara et al., 2017). Thus, significant scope exists for enhancing nutrient use efficiency in onion cultivation by aligning fertilizer application with crop demand. However, onions, being shallow-rooted and heavy-feeding crops, often face challenges in nutrient uptake, especially when nutrients leach beyond the root zone, rendering them unavailable to the plants (Thangasamy, 2016). To address this issue, applying fertilizer nutrients in reduced amount directly to the root zone or via microbial inoculation has shown promise in enhancing nutrient use efficiency (Shahwar et al., 2023).

The application of plant growth-promoting rhizobacteria, endophytic fungi, or vesicular arbuscular mycorrhiza (VAM) along with mineral fertilizers enhances plant growth and crop yield through phytohormone secretion, nutrient supplementation, and pathogen suppression (Zhang et al., 2021; Yu et al., 2024). Mycorrhizal inoculants such as *Glomus mosseae* have been found to enhance growth and yield, particularly in phosphorus (P)-deficient soil conditions (Chen et al., 2017). Given the shallow root system of onions, inoculation with these endophytic fungi or VAM may enhance P uptake (Augé and Moore, 2005). The hyphae of these microbes serve as extensions of the roots, aiding in the extraction of water and nutrients from beyond root zones as well as from the deeper soil layers, thereby increasing nutrient uptake and crop yield (Bucking et al., 2012). Furthermore, phosphorus-solubilizing bacteria (PSB) play a crucial role in converting fixed or unavailable nutrients into forms that are readily available to plants (Wang et al., 2024; Xu et al., 2024). A recent study by Novello et al. (2021) reported that the inoculation with PSB resulted in increased plant growth and nutrient concentration in onions. Likewise, *Serendipita indica (S. indica)* is a beneficial endosymbiont known for enhancing plant growth, development, induction of stress tolerance, and nutrient acquisition through different modes of action (Gill et al., 2016; Saleem et al., 2022; Roylawar et al., 2023).

To date, several studies have focused on selecting the suitable microorganisms for establishing successful symbiotic relationships in various environmental conditions and farming systems (Bolandnazar, 2009; Albrechtova et al., 2012; Caruso et al., 2018). Furthermore, numerous studies have focused on the implications of these ecological associations on onion plant performance, particularly concerning bulb yield and quality (Shinde and Shinde, 2016; Fredotovic and Puizina, 2019; Petrovic et al., 2020). Despite the potential benefits, *S. indica* has not been widely used as a biofertilizer in onion cultivation, while VAM and PSB have yielded variable results.

Therefore, the following hypotheses were formulated: 1. The application of mineral fertilizers along with PGPM will enhance onion plant growth, yield, and nutrient uptake compared to the use of mineral fertilizers alone, and 2. The combined use of PGPM and mineral fertilizers will result in better post-harvest soil fertility, maintaining or improving soil nutrient levels and health compared to the use of mineral fertilizers alone. To test these hypotheses, a field experiment was conducted with the objective to evaluate the effect of different fertilizer levels and PGPM (such as *Serendipita indica*, *Bacillus megaterium*, *Paenibacillus polymyxa, Bacillus* sp., *Glomus fasciculum*, *G. intraradices*, *Aculospora* sp., and *Gigaspora* sp.) inoculations on plant growth parameters, onion yield, nutrient uptake, and post-harvest soil fertility to provide insights for improving onion productivity.

## Materials and methods

### Experimental site

A two-year field experiment during 2018–2019 and 2019–2020 was conducted at the experimental farm of the Indian Council of Agricultural Research – Directorate of Onion and Garlic Research (ICAR–DOGR) in Pune, Maharashtra, India. The experimental site was situated at coordinates 18.32° N and 73.51° E, at an elevation of 645 meters above mean sea level (MSL). The climatic conditions of the experimental site were characterized by a tropical, dry humid climate with a mean annual precipitation of 820 mm. Throughout the cultivation period, the maximum air temperature ranged from 28.9 to 36.8°C, while the minimum air temperature varied from 9.7 to 17.2°C. The soils in the experimental field were classified as clay loam, and the initial soil analysis conducted indicated low available N and medium soil organic carbon status (Table 1).

**Table 1:** Values of soil properties recorded pre-planting (mean of two years values with standard error).

Soil properties	Initial value
Soil pH	8.10 ± 0.11
Electrical conductivity (dS m^−1^)	0.18 ± 0.01
Soil organic carbon (g kg^−1^)	6.78 ± 0.05
Soil available N (mg kg^−1^)	96.1 ± 3.0
Soil available P (mg kg^−1^)	8.99 ± 0.39
Soil available K (mg kg^−1^)	212.7 ± 6.7
Soil available S (mg kg^−1^)	7.40 ± 0.22

### Experimental details

The field experiment was designed using a completely randomized block design with nine treatments. The treatments included: T1: Control (without fertilizers), T2: 50% recommended dose of fertilizer (RDF) + PSB consortia (PSB), T3: 50% RDF + VAM consortia (VAM), T4: 50% RDF + *S. indica*, T5: 50% RDF alone, T6: 100% RDF + PSB, T7: 100% RDF + VAM, T8: 100% RDF + *S. indica*, T9: 100% RDF alone. The PSB consortia consisted of *Bacillus megaterium*, *Paenibacillus polymyxa,* and other *Bacillus* sp., while the VAM consortia consisted of *Glomus fasciculum*, *G. intraradices, Acaulospora* sp., and *Gigaspora* sp. Each treatment was replicated three times. Onion cv. Bhima Shakti was sown in the nursery during the second week of October in both years. Simultaneously, the main field was prepared by ploughing using the mold board plough and tilled using the cultivator. Raised beds of 1.2 m in width and 14 m in length were prepared after pulverizing the soil with a rotavator. Organic manures were applied at a rate of 5 t ha^−1^ to all treatments except the control plot. The pre-emergence herbicide oxyfluorfen was applied 7 days before transplanting to control weeds, followed by irrigation. Before transplanting, 100% of the required phosphorus (P), potassium (K), and sulfur (S), along with 20% of nitrogen (N), were applied as basal fertilizer. Mineral fertilizers, including 10:26:26, muriate of potash, and bentonite S, were used to supply N, P, K, and S. Forty-five-day-old seedlings were transplanted at a spacing of 15 cm between rows and 10 cm between plants during the third week of December in both years. The plot size for each treatment was 16.8 m^2^. Before transplanting, a slurry of *S. indica*, PSB, and VAM was prepared, and seedling roots were immersed in the slurry for 2 h before transplanting. After treatment, the slurry with microbes was applied to the respective plots. The remaining 80% of N was applied through urea in three equal splits at 15, 30, and 45 days after transplanting (DAT). Irrigation water was applied as required through the drip system. Weeds were manually removed at 45 DAT, and all other intercultural operations and plant protection measures were carried out at timely intervals as per the ICAR-DOGR standard package of practices. Twenty-four plants were labeled and measured for plant growth parameters, including plant height and number of leaves, at 30 and 45 DAT in each plot. Additionally, twenty-four fully matured leaves were collected from each plot to determine the leaf area index. Onion bulbs were harvested in the second week of April after the crop exhibited 50% of the top fall. Three days after field curing, the bulbs were separated, leaving a 2.5 cm neck, and the bulb yield was recorded and expressed in tonnes per hectare (t ha^−1^).

### Soil sampling and analysis

Soil samples were collected from all treatments at a depth of 0−30 cm after harvesting. These soil samples were processed and sieved using a 2.0 mm sieve before being used for soil analysis. Standard protocols were followed to analyze soil pH, electrical conductivity, soil organic carbon, and the concentrations of available N, P, K, and S. A soil water suspension with a ratio of 1:2 was prepared, and soil pH and electrical conductivity were measured using a pH meter and conductivity bridge, respectively. Soil organic carbon (SOC) was determined using the wet-oxidation method described by Walkley and Black (1934). The available soil N was estimated using the alkaline permanganate method, P by using Olsen’s method, K by the 1 N ammonium acetate method, and S by the 0.15 M CaCl_2_ extraction method (Jackson, 1967).

### Plant sampling and analysis

Twenty-four plant samples (whole plants) were collected from each treatment at the time of harvest. These samples were thoroughly washed and rinsed with distilled water. The bulbs and leaves were then separated, chopped into pieces, and air-dried. Once air-dried, the bulb and leaf samples were further over-dried in an oven at 58°C until a constant weight was reached. After reaching a constant weight, the dry weight of both the bulbs and leaves was recorded. Subsequently, the leaf and bulb samples were ground, passed through a 2.0 mm sieve, and used for plant nutrient analysis. Total N was analyzed using the micro-Kjeldahl method. To estimate total P, K, and S (S), 0.5 g of plant samples were digested using di-acid. Following digestion, the digest was thoroughly washed with distilled water and filtered through Whatman Number 40 filter paper. Subsequently, the filtrate was used for total P, K, and S analyses. Total P was determined using the ammonium vanado-molybdate method, total K using the flame photometer method, and total S using the turbidimetric method (Jackson, 1967). The nutrient recovery efficiency (%), which represents the quantity of nutrient absorbed per unit of nutrient applied, was computed using the formula: Nutrient recovery efficiency (%) = (U_n_-U_0_)/n × 100, where U_n_ represents the nutrient uptake by the crop with the application of N, P, K, and S fertilizers, U_0_ signifies the nutrient uptake by the crop without fertilization, and n stands for the quantity of fertilizer applied (Dobermann, 2007).

### Statistical analysis

Plant growth parameters, nutrient concentrations, onion yield, dry matter accumulation, nutrient uptake, and nutrient recovery efficiency data were analyzed using the two-way ANOVA in R software version 4.3.3 (R Core Team, 2024). A *post hoc* analysis with the least significant difference was conducted to compare means following the ANOVA. Subsequently, Pearson’s correlation coefficient was calculated to assess the associations between various traits. Furthermore, principal component analysis (PCA) and biplot PCA were employed to elucidate the relationships among treatments and parameters. These analyses were used to illustrate the interrelationships among the tested treatments based on different parameters.

## Results

### Plant growth parameters

The fertilizer treatments and the year of cultivation did not result in a significant increase in the number of leaves (Table 2). However, both the number of leaves and plant height increased with the progression of crop age from 30 to 60 DAT. Specifically, the number of leaves and plant height increased by 17.4 to 28.8% and 21.2 to 25.6%, respectively, compared to values recorded at 30 DAT. Notably, during the 2019–2020 season, the fertilizer treatments resulted in significantly higher plant height compared to the control treatment. Although there were no differences with statistical significance among the fertilizer treatments, the combination of 100% RDF with *S. indica* led to the highest plant height compared to other treatments. The increase in plant height ranged from 5.4 to 5.6%, compared to 100% RDF without microbial inoculation. Additionally, across all treatments, the highest values for the number of leaves and plant height were observed in the year 2019–2020 compared to 2018–2019.

**Table 2:** Effect of mineral fertilizers and microbial inoculations on plant growth parameters.

Treatment	Number of leaves			Plant height (cm)				Leaf area index		
	2019–20		2020–21		2019–20		2020–21		2020–21	
	30 DAT	60 DAT	30 DAT	60 DAT	30 DAT	60 DAT	30 DAT	60 DAT	30 DAT	60 DAT
Control	6.0 ^a^	7.7 ^a^	6.3 ^a^	7.3 ^a^	41.3 ^a^	50.6 ^a^	38.0 ^a^	44.6 ^b^	0.53 ^c^	1.46 ^c^
50% RDF + PSB	6.0 ^a^	7.3 ^a^	7.0 ^a^	8.0 ^a^	42.8 ^a^	51.8 ^a^	48.9 ^a^	56.5 ^a^	0.64 ^ab^	1.77 ^b^
50% RDF + VAM	5.7 ^a^	7.0 ^a^	7.0 ^a^	7.7 ^a^	43.1 ^a^	54.5 ^a^	46.0 ^a^	54.3 ^a^	0.62 ^ab^	1.72 ^b^
50% RDF + *S. indica*	5.7 ^a^	7.7 ^a^	6.7 ^a^	8.0 ^a^	44.8 ^a^	54.3 ^a^	48.0 ^a^	56.7 ^a^	0.66 ^a^	1.77 ^b^
50% RDF	5.7 ^a^	7.3 ^a^	7.0 ^a^	8.3 ^a^	41.3 ^a^	51.2 ^a^	47.7 ^a^	54.3 ^a^	0.56 ^bc^	1.70 ^bc^
100% RDF + PSB	6.0 ^a^	8.0 ^a^	7.0 ^a^	8.3 ^a^	44.4 ^a^	55.1 ^a^	43.9 ^a^	57.4 ^a^	0.65 ^a^	2.12 ^a^
100% RDF + VAM	6.0 ^a^	7.7 ^a^	6.7 ^a^	8.7 ^a^	44.2 ^a^	56.9 ^a^	45.0 ^a^	57.4 ^a^	0.59 ^abc^	2.05 ^a^
100% RDF + *S. indica*	6.0 ^a^	8.0 ^a^	7.0 ^a^	8.3 ^a^	45.0 ^a^	57.8 ^a^	47.2 ^a^	60.2 ^a^	0.65 ^a^	2.17 ^a^
100% RDF	5.7 ^a^	7.3 ^a^	7.0 ^a^	8.3	44.1 ^a^	58.8 ^a^	44.8 ^a^	57.0 ^a^	0.64 ^ab^	2.02 ^a^
Mean	5.9	7.6	6.9	8.1	43.4	54.5	45.5	55.4	0.62	1.87
SEM±	0.2	0.3	0.2	0.4	1.5	2.2	2.1	1.4	0.02	0.05
CV%	6.3	5.8	4.8	7.4	5.9	7.0	7.8	4.4	4.69	4.55
*p* = 0.05	0.70	0.17	0.19	0.28	0.54	0.16	0.06	<0.0001	<0.0001	<0.0001

Values with different letters in the same year indicate significant differences (*p* < 0.05) as assessed using the least significant difference (LSD) test.

All microbial inoculation treatments exhibited a significant increase in leaf area index at both 30 and 60 DAT during the year 2019–2020. Specifically, inoculation with *S. indica* at either 50% or 100% RDF led to a significant increase in leaf area index at both 30 and 60 DAT. The application of 100% RDF, whether with or without microbial inoculation, resulted in a significant increase in leaf area index at 60 DAT, surpassing that of the 50% RDF treatments, with or without microbial inoculation, as well as the control treatment. Although the leaf area index was statistically comparable to that of 100% RDF, the treatment receiving 100% RDF with *S. indica* inoculation exhibited a 7.4% increase compared to 100% RDF alone. Additionally, the roots of onion plants were examined for colonization at 45 DAT using staining techniques, confirming the presence of *S. indica* colonization.

### Nutrient concentrations

Leaves generally exhibited higher concentrations of K and lower concentrations of N, P, and S compared to bulbs in both experimental years (Table 3). The fertilizer treatments significantly influenced the concentrations of N, P, K, and S in both leaves and bulbs. The treatment receiving 100% RDF, either alone or in combination with *S. indica* or PSB inoculation, recorded the highest concentrations of N, P, K, and S in both leaves and bulbs. Specifically, the application of 100% RDF along with *S. indica* increased N concentrations in leaves by 7.3–19.5%, P by 42.9–43.8%, K by 1.8–2.8%, and S by 6.7%. In bulbs, it increased N concentrations by 22.9%, P by 8.6%, K by 12.5–12.8%, and S by 8.9–10.6% compared to 100% RDF alone. These values were notably higher than those observed in the treatments receiving 50% RDF alone or in combination with PSB, *S. indica*, or VAM inoculation, as well as the control. In terms of yearly variations, concentrations of N, P, K, and S in bulbs and N and K in leaves were higher in the year 2018–2019 compared to the values recorded in the year 2019–2020. Conversely, P and S concentrations in leaves were higher in 2019–2020 compared to those observed in 2018–2019.

**Table 3:** Effect of mineral fertilizer and microbial inoculation on nutrient concentration.

Treatments	Nitrogen concentration (%)				Phosphorus concentration (%)				Potassium concentration (%)				Sulphur concentration (%)			
	2018–19		2019–20		2018–19		2019–20		2018–19		2019–20		2018–19		2019–20	
	Leaves	Bulbs	Leaves	Bulbs	Leaves	Bulbs	Leaves	Bulbs	Leaves	Bulbs	Leaves	Bulbs	Leaves	Bulbs	Leaves	Bulbs
Control	1.02 ^e^	1.18 ^d^	0.44 ^d^	0.72 ^b^	0.09 ^a^	0.29 ^c^	0.34 ^cd^	0.23 ^de^	1.55 ^bc^	1.01 ^c^	1.30 ^a^	0.87 ^a^	0.21 ^a^	0.44 ^ab^	0.33 ^c^	0.32 ^b^
50% RDF + PSB	1.31 ^bc^	1.93 ^ab^	1.12 ^bc^	1.32 ^a^	0.09 ^a^	0.36 ^ab^	0.35 ^cd^	0.26 ^de^	1.60 ^abc^	1.39 ^ab^	1.08 ^b^	0.78 ^abc^	0.23 ^a^	0.48 ^ab^	0.57 ^a^	0.47 ^a^
50% RDF + VAM	1.28 ^c^	1.57 ^c^	1.29 ^a^	1.12 ^ab^	0.07 ^a^	0.34 ^ab^	0.44 ^abc^	0.30 ^cd^	1.80 ^a^	1.15 ^bc^	1.03 ^b^	0.73 ^bc^	0.20 ^a^	0.43 ^b^	0.48 ^ab^	0.50 ^a^
50% RDF + *S. indica*	1.41 ^ab^	1.83 ^bc^	1.10 ^bc^	1.07 ^ab^	0.09 ^a^	0.38 ^ab^	0.43 ^bc^	0.26 ^de^	1.45 ^c^	1.32 ^ab^	1.03 ^b^	0.73 ^bc^	0.20 ^a^	0.46 ^ab^	0.53 ^ab^	0.50 ^a^
50% RDF	1.12 ^de^	1.83 ^bc^	0.98 ^c^	1.05 ^ab^	0.09 ^a^	0.33 ^bc^	0.26 ^d^	0.22 ^e^	1.52 ^bc^	1.23 ^abc^	0.99 ^b^	0.69 ^c^	0.22 ^a^	0.41 ^b^	0.45 ^ab^	0.47 ^a^
100% RDF + PSB	1.30 ^bc^	2.02 ^ab^	1.06 ^bc^	1.29 ^a^	0.06 ^a^	0.37 ^ab^	0.32 ^d^	0.37 ^abc^	1.62 ^abc^	1.38 ^ab^	1.17 ^ab^	0.87 ^a^	0.18 ^a^	0.56 ^a^	0.44 ^bc^	0.43 ^a^
100% RDF + VAM	1.29 ^bc^	2.05 ^ab^	1.08 ^bc^	1.39 ^a^	0.09 ^a^	0.36 ^ab^	0.53 ^a^	0.43 ^a^	1.67 ^ab^	1.37 ^ab^	1.12 ^ab^	0.84 ^ab^	0.12 ^a^	0.47 ^ab^	0.50 ^ab^	0.44 ^a^
100% RDF + *S. indica*	1.47 ^a^	2.05 ^ab^	1.17 ^ab^	1.34 ^a^	0.10 ^a^	0.38 ^a^	0.46 ^ab^	0.38 ^ab^	1.71 ^ab^	1.44 ^a^	1.11 ^ab^	0.88 ^a^	0.21 ^a^	0.52 ^ab^	0.48 ^ab^	0.49 ^a^
100% RDF	1.23 ^cd^	2.20 ^a^	1.09b ^c^	1.09 ^ab^	0.07 ^a^	0.38 ^a^	0.32 ^d^	0.35 ^bc^	1.68 ^ab^	1.28 ^abc^	1.08 ^b^	0.78 ^abc^	0.22a	0.47 ^ab^	0.45 ^ab^	0.45 ^a^
Mean	1.27	1.85	1.04	1.16	0.08	0.35	0.38	0.31	1.62	1.29	1.1	0.8	0.2	0.47	0.47	0.45
SEM±	0.03	0.06	0.05	0.08	0.01	0.01	0.02	0.02	0.04	0.06	0.04	0.03	0.03	0.03	0.02	0.02
CV%	3.42	5.78	8.56	12.22	25.06	5.11	9.01	8.1	4.44	7.37	5.97	5.88	21.81	9.17	8.57	6.58
P = 0.05	<0.0001	<0.0001	<0.0001	0.001	0.477	<0.0001	<0.0001	<0.0001	0.001	0.001	0.001	0.001	0.168	0.018	<0.0001	<0.0001

Values with different letters in the same year indicate significant differences (*p* < 0.05) as assessed using the LSD test.

### Nutrient uptake

The application of 100% RDF in combination with microbial inoculation significantly increased the total N, P, K, and S uptake compared to treatments involving 50% RDF with or without microbial inoculation and the absolute control in both experimental years (Table 4). The highest uptake of N, P, K, and S was observed in the treatment receiving 100% RDF along with *S. indica* in both years, closely followed by the treatments with 100% RDF along with PSB. The application of 100% RDF with *S. indica* led to the highest N, P, K, and S uptake, showing an increase of 6.9–29.9% for N, 13.7–21.7% for P, 20.0–23.7% for K, and 18.1–23.4% for S compared to the treatment with 100% RDF alone. Similarly, the application of 100% RDF with PSB inoculation increased N, P, K, and S uptake by 4.0–28.1%, 8.3–19.6%, 17.6–22.9%, and 7.0–31.4%, respectively, compared to the treatment with 100% RDF alone. The highest mean total N and K uptake was recorded in the year 2018–2019, whereas the highest mean total P and S uptake was recorded in the year 2019–2020. N, P, K, and S recovery efficiency was notably higher in treatments inoculated with either *S. indica* or PSB compared to those treated with mineral fertilizers alone (Table 5). Furthermore, nutrient uptake efficiencies in these treatments exceeded with those observed in treatments receiving mineral fertilizers alone or in combination with VAM inoculation.

**Table 4:** Effect of mineral fertilizer application and microbial inoculation on total nutrient uptake by onion.

Treatment	Total N uptake (kg ha^−1^)		Total P uptake (kg ha^−1^)		Total K uptake (kg ha^−1^)		Total S uptake (kg ha^−1^)	
	2018–19	2019–20	2018–19	2019–20	2018–19	2019–20	2018–19	2019–20
Control	58.4 ^c^	37.1 ^c^	13.17 ^d^	13.36 ^d^	54.5 ^e^	49.6d ^e^	20.83 ^c^	17.30 ^c^
50% RDF + PSB	95.9 ^b^	118.9 ^ab^	16.22 ^cd^	24.33 ^c^	74.9 ^bcd^	73.6 ^bc^	22.80 ^c^	44.10 ^ab^
50% RDF + VAM	84.6 ^b^	85.8 ^b^	16.47 ^bcd^	23.92 ^c^	70.1 ^d^	57.2 ^de^	21.87 ^c^	37.50 ^b^
50% RDF + *S. indica*	99.9 ^b^	96.8 ^ab^	18.82 ^abc^	24.45 ^c^	75.8 ^bcd^	67.9 ^cd^	23.80 ^c^	44.93 ^ab^
50% RDF	93.8 ^b^	89.1 ^b^	16.00 ^cd^	19.40 ^d^	68.9 ^de^	61.9 ^cde^	20.83 ^c^	40.33 ^ab^
100% RDF + PSB	126.2 ^a^	126.3 ^a^	21.43 ^a^	36.64 ^a^	93.4 ^ab^	89.7 ^a^	33.47 ^a^	42.93 ^ab^
100% RDF + VAM	123.3 ^a^	116.4 ^ab^	20.54 ^ab^	38.06 ^a^	90.0 ^abc^	74.4 ^abc^	26.83 ^bc^	38.33 ^b^
100% RDF + *S. indica*	129.8 ^a^	128.1 ^a^	22.48 ^a^	37.28 ^a^	98.2 ^a^	87.6 ^ab^	31.43 ^ab^	47.40 ^a^
100% RDF	121.4 ^a^	98.6 ^ab^	19.78 ^abc^	30.64 ^b^	79.4 ^bcd^	73.0 ^bc^	25.47 ^bc^	40.13 ^ab^
Mean	103.4	96.7	18.32	27.56	78.3	70.5	25.26	39.22
SEM±	3.4	7.2	0.81	0.96	3.0	3.1	1.32	1.60
CV%	5.7	12.4	7.65	6.02	6.1	7.6	9.01	7.08
P = 0.05	<0.0001	<0.0001	<0.0001	<0.0001	<0.0001	<0.0001	<0.0001	<0.0001

Values with different letters in the same year indicate significant differences (*p* < 0.05) as assessed using the LSD test.

**Table 5:** Influence of mineral fertilizers and microbial inoculation on nutrient recovery efficiency.

Treatments	N recovery efficiency (%)		P recovery efficiency (%)		P recovery efficiency (%)		S recovery efficiency (%)	
	2018–19	2019–20	2018–19	2019–20	2018–19	2019–20	2018–19	2019–20
50% RDF + PSB	68.2 ^a^	148.6 ^a^	15.2 ^b^	55.3 ^a^	68.2 ^a^	80.0 ^a^	13.0 ^bc^	178.5 ^ab^
50% RDF + VAM	47.7 ^b^	88.4b ^cd^	16.5 ^b^	53.3 ^a^	52.2 ^ab^	25.3 ^c^	6.8 ^c^	134.8 ^c^
50% RDF + *S. indica*	75.4 ^a^	108.4 ^b^	28.2 ^a^	55.9 ^a^	71.0 ^a^	61.1 ^ab^	19.6 ^abc^	184.4 ^a^
50% RDF	64.4 ^ab^	94.5 ^bc^	14.1 ^b^	30.7 ^c^	48.0 ^ab^	40.0 ^bc^	−0.3 ^c^	153.6 ^bc^
100% RDF + PSB	61.7 ^ab^	81.0 ^bcd^	20.6 ^ab^	58.4 ^a^	64.8 ^ab^	66.8 ^a^	42.0 ^a^	85.3 ^de^
100% RDF + VAM	59.0 ^ab^	72.1 ^cd^	18.4 ^ab^	62.0 ^a^	59.1 ^ab^	41.3 ^bc^	19.9 ^abc^	70.1 ^e^
100% RDF + *S. indica*	64.9 ^ab^	82.7 ^bcd^	23.3 ^ab^	60.1 ^a^	72.9 ^a^	63.3 ^ab^	35.3 ^ab^	100.4 ^d^
100% RDF	57.3 ^ab^	55.9 ^d^	16.5 ^b^	43.6 ^b^	41.6 ^b^	38.9 ^bc^	15.4 ^bc^	76.1 ^de^

Values with different letters in the same year indicate significant differences (*p* < 0.05) as assessed using the LSD test.

### Dry matter yield

Dry matter accumulation in leaves, bulbs, and total dry matter yield responded to both fertilizer treatments and the cultivation year (Table 6). The highest leaf dry matter yield was recorded in treatments receiving *S. indica* inoculation with 100% RDF during the year 2018–2019, which was comparable to treatments receiving 100% RDF with VAM inoculation. In the subsequent year, the leaf dry matter yield was found to be significantly higher in treatments involving PSB inoculation and 100% RDF, which was statistically on par with the treatment receiving 100% RDF alone. For bulb dry matter yield and total plant dry matter yield in both experimental years, the maximum values were observed in treatments receiving 100% RDF with PSB, which was closely followed by treatments receiving 100% RDF with *S. indica* inoculation. The treatment receiving 100% RDF with PSB inoculation increased total dry matter yield by 11.6 and 10.5% in the year 2018–2019 and 2019–2020, respectively, compared to that of 100% RDF alone. Meanwhile, the application of 100% RDF with *S. indica* inoculation increased total dry matter yield by 11.5 and 7.6% in 2018–2019 and 2019–2020, respectively, compared to the treatment receiving 100% RDF alone. Leaf dry matter yield exhibited no significant difference between the two years, while the bulb and total dry matter yield recorded in 2019–2020 were significantly higher than those documented in the previous year 2018–2019.

**Table 6:** Influence of mineral fertilizer and microbial inoculation on dry matter yield.

Treatment	Leaf dry matter yield (kg ha^−1^)		Bulb dry matter yield (kg ha^−1^)		Dry matter yield (kg ha^−1^)	
	2018–19	2019–20	2018–19	2019–20	2018–19	2019–20
Control	647 ^b^	613 ^c^	4,398 ^c^	4,813 ^d^	5,046 ^e^	5,426 ^d^
50% RDF + PSB	913 ^a^	898 ^ab^	4,333 ^c^	8,234 ^ab^	5,246 ^de^	9,132 ^ab^
50% RDF + VAM	926 ^a^	752 ^bc^	4,639 ^bc^	6,778 ^c^	5,565 ^cd^	7,530 ^c^
50% RDF + *S. indica*	875 ^ab^	814 ^abc^	4,778 ^abc^	8,171 ^ab^	5,653 ^cd^	8,985 ^ab^
50% RDF	768 ^ab^	775 ^bc^	4,648 ^bc^	7,783 ^abc^	5,416 ^de^	8,558 ^bc^
100% RDF + PSB	989 ^a^	1,021 ^a^	5,630 ^a^	8,929 ^a^	6,618 ^a^	9,950 ^a^
100% RDF + VAM	938 ^a^	914 ^ab^	5,426 ^ab^	7,680 ^bc^	6,363 ^ab^	8,594 ^bc^
100% RDF + *S. indica*	1,011 ^a^	960 ^ab^	5,602 ^a^	8,727 ^ab^	6,613 ^a^	9,687 ^ab^
100% RDF	942 ^a^	1,028 ^a^	4,991 ^abc^	7,978 ^abc^	5,933 ^bc^	9,006 ^ab^
Mean	890	864	4,938	7,677	5,828	8,541
SEM±	49	48	171	244	171	252
CV%	9.5	9.6	6.1	5.5	5.1	5.1
P = 0.05	0.002	<0.0001	<0.0001	<0.0001	<0.0001	<0.0001

Values with different letters in the same year indicate significant differences (*p* < 0.05) as assessed using the least significant difference (LSD) test.

### Total onion yield

**Figure 1:**
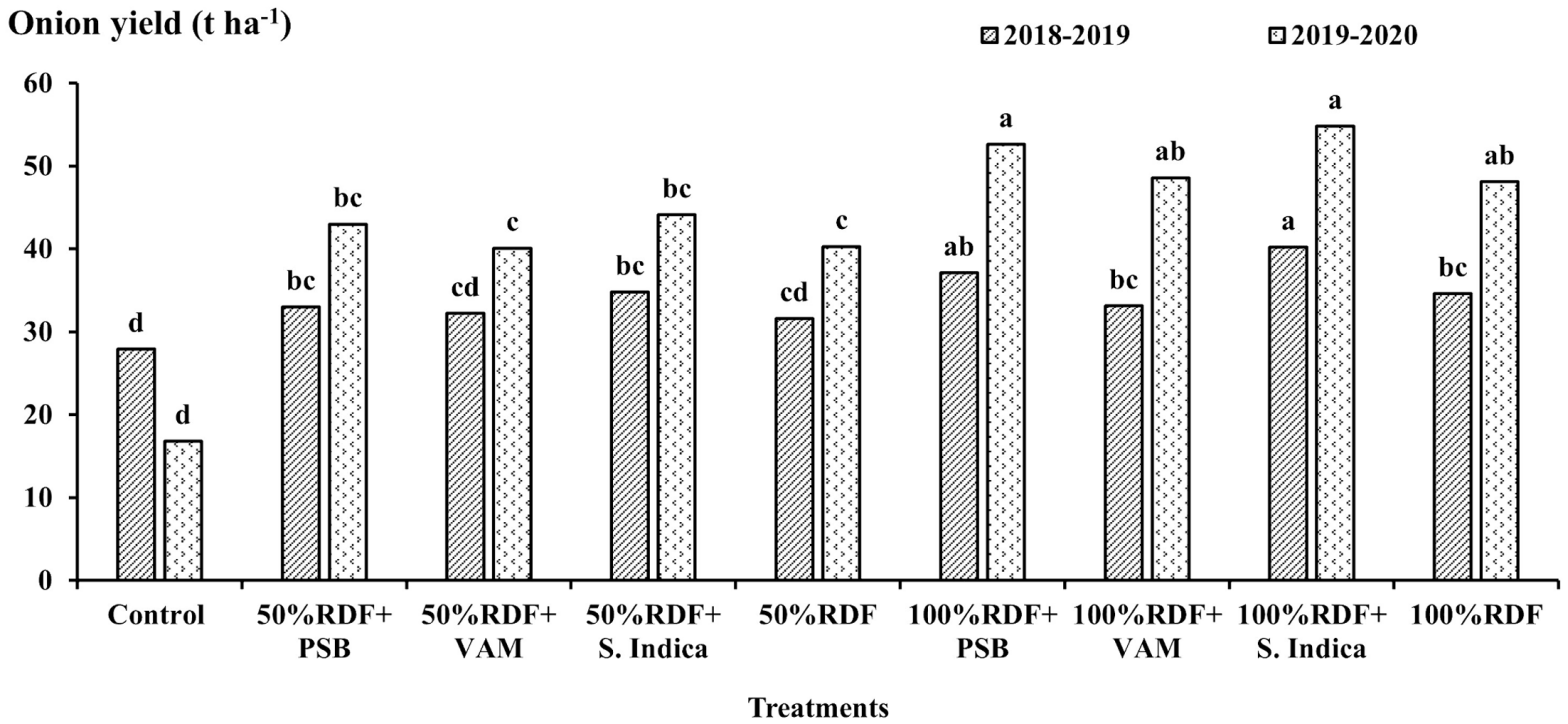
Effect of application of mineral fertilizers and microbial inoculation on onion yield (t ha^−1^). Values with different letters in the same year indicate significant differences (*p* < 0.05) as assessed using the LSD test.

The onion bulb yield exhibited a significant increase when inoculated with *S. indica* or PSB in combination with a 100% mineral fertilizer application (Figure 1). This increase was significantly higher in comparison with the onion yield observed in treatments receiving only 50% RDF and the absolute control in both the years 2018–2019 and 2019–2020. Furthermore, the combination of *S. indica* inoculation with 100% RDF fertilizer application produced the highest total bulb yield, which was closely followed by the treatment involving 100% RDF with PSB inoculation during both experimental years. In 2018–2019, the application of 100% RDF along with *S. indica* or PSB significantly increased the total bulb yield compared to 100% RDF alone. However, in 2019–2020, these treatments produced yields that were statistically comparable to 100% RDF alone. The combination of 100% RDF with *S. indica* resulted in yield increases of 16.2% in 2018–2019 and 13.9% in 2019–2020 compared to 100% RDF alone. Similarly, the application of 100% RDF along with PSB inoculation led to an increase in bulb yield of 7.2 and 9.4% in the respective years of the onion crop. Contrarily, treatments involving 50% RDF with microbial inoculation (*S. indica*, PSB, or VAM) and VAM inoculation with 100% RDF did not exhibit a considerable increase in bulb yield compared to the treatments receiving 100% mineral fertilizer in both years. Additionally, there was a significant enhancement in onion yield observed with the progressive increase in fertilizer levels from 50% RDF to 100% RDF. During these experimental years, a higher yield was registered in 2019–2020 when compared to the previous crop year 2018–2019.

### Post-harvest soil properties

The application of mineral fertilizers, either alone or in combination with microbial inoculations, did not have a significant influence on post-harvest soil properties (Table 7). Notably, soil available N concentration was 24.7% higher in 2018–2019 compared to 2019–2020. In contrast, the concentrations of soil available P, K, and S were higher in 2019–2020 than in 2018–2019, with increases of 33.1, 25.9, and 9.9%, respectively, compared to the values recorded in 2018–2019.

**Table 7:** Influence of application of mineral fertilizers and microbial inoculation on post-harvest soil fertility status.

Treatment	Soil pH		EC (dS m^−1^)		SOC (g kg^−1^)		Soil available nutrients (mg kg^−1^)							
							N		P	K			S	
	2018–19	2019–20	2018–19	2019–20	2018–19	2019–20	2018–19	2019–20	2018–19	2019–20	2018–19	2019–20	2018–19	2019–20
Control	7.81 ^a^	7.59 ^ab^	0.19 ^a^	0.19 ^ab^	6.83 ^a^	6.47 ^a^	90.6 ^b^	68.9 ^a^	9.47 ^a^	12.78 ^a^	240.3 ^a^	289.2 ^a^	14.32 ^a^	14.38 ^a^
50% RDF + PSB	7.78 ^a^	7.63 ^ab^	0.21 ^a^	0.17 ^b^	6.80 ^a^	6.90 ^a^	99.9 ^ab^	75.1 ^a^	10.04 ^a^	13.90 ^a^	233.9 ^ab^	287.8 ^a^	14.86 ^a^	16.42 ^a^
50% RDF + VAM	7.81 ^a^	7.63 ^ab^	0.21 ^a^	0.19 ^ab^	6.97 ^a^	6.37 ^a^	105.4 ^a^	69.4 ^a^	9.57 ^a^	13.58 ^a^	223.5 ^ab^	287.8 ^a^	14.90 ^a^	16.16 ^a^
50% RDF + *S. indica*	7.94 ^a^	7.55 ^ab^	0.20 ^a^	0.20 ^ab^	7.03 ^a^	6.33 ^a^	99.9 ^ab^	70.5 ^a^	9.69 ^a^	12.59 ^a^	224.0 ^ab^	282.5 ^a^	16.53 ^a^	18.06 ^a^
50% RDF	7.84 ^a^	7.70 ^ab^	0.18 ^a^	0.19 ^ab^	6.53 ^a^	6.63 ^a^	88.8 ^b^	70.5 ^a^	10.23 ^a^	12.07 ^a^	222.5 ^a^	290.1 ^a^	14.58 ^a^	16.18 ^a^
100% RDF + PSB	7.84 ^a^	7.80 ^a^	0.20 ^a^	0.25 ^b^	6.40 ^a^	6.57 ^a^	88.8 ^b^	73.9 ^a^	9.54 ^a^	12.13 ^a^	218.0 ^b^	286.0 ^a^	13.96 ^a^	18.32 ^a^
100% RDF + VAM	7.79 ^a^	7.50 ^b^	0.18 ^a^	0.24 ^ab^	6.77 ^a^	7.17 ^a^	94.3 ^ab^	73.9 ^a^	8.18 ^a^	11.69 ^a^	221.0 ^b^	279.2 ^a^	14.84 ^a^	17.03 ^a^
100% RDF + *S. indica*	7.84 ^a^	7.51 ^b^	0.20 ^a^	0.28 ^a^	6.70 ^a^	6.57 ^a^	94.3 ^ab^	70.9 ^a^	10.11 ^a^	12.72 ^a^	227.5 ^ab^	280.7 ^a^	15.51 ^a^	16.94 ^a^
100% RDF	7.73 ^a^	7.54 ^ab^	0.18 ^a^	0.22 ^ab^	6.80 ^a^	6.90 ^a^	95.4 ^ab^	72.2 ^a^	9.69 ^a^	13.64 ^a^	226.0 ^ab^	277.9 ^a^	17.64 ^a^	17.14 ^a^
Mean	7.82	7.61	0.20	0.20	6.76	6.66	95.3	71.7	9.61	12.79	226.0	284.6	15.24	16.74
SEM±	0.07	0.06	0.01	0.02	0.25	0.24	2.84	2.87	3.25	1.14	3.42	6.41	0.82	0.47
CV%	1.55	1.25	6.01	15.29	6.41	6.15	5.16	6.94	7.85	15.38	2.61	3.90	9.35	12.04

Values with different letters in the same year indicate significant differences (*p* < 0.05) as assessed using the LSD test.

### Principle component analysis

**Figure 2:**
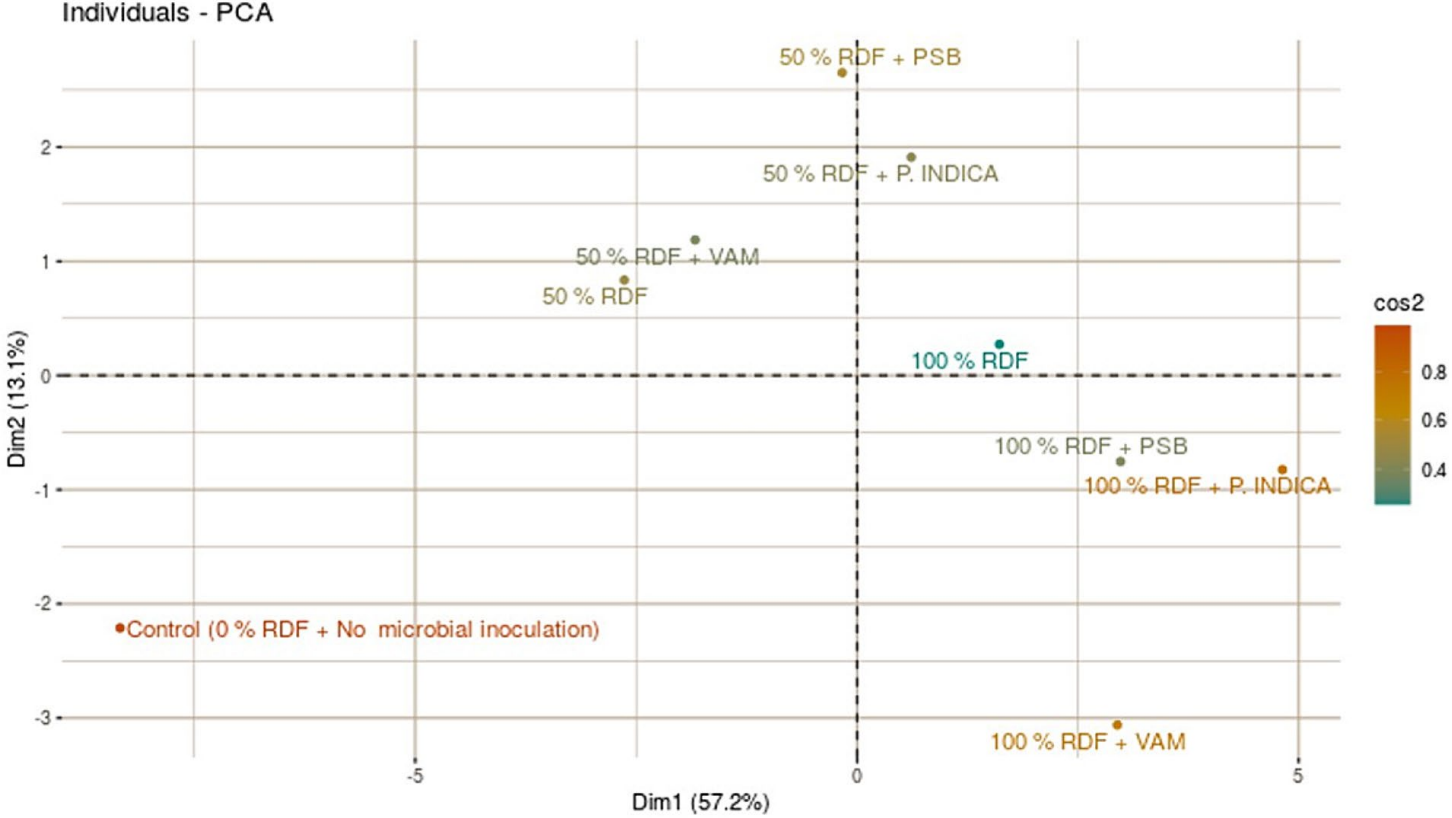
Principal component analysis (PCA) showing the effects of mineral fertilizer application and microbial inoculation on various parameters in onion.

PCA showed a strong relationship between various parameters and treatments, as illustrated by the principal component biplots (Figure 2). The two-way matrix between treatment and parameter biplots showed that a narrower angle between different parameters in the same direction signified a strong association between those parameters in classifying treatments. Out of nine treatments assessed in the present study, the optimal treatments, specifically 100% RDF with either *S. indica* or PSB, were positioned closer to and aligned along the vector line direction.

## Discussion

In a two-year study, the effects of VAM, PSB, and *S. indica* inoculation with 50 and 100% RDF on onion growth, yield, nutrient uptake, and soil fertility were examined.

The application of *S. indica* or PSB with 100% RDF led to notable increases in various plant growth parameters such as plant height, number of leaves, and leaf area index, along with increased biomass production, and onion yield. This increase is potentially attributable to enhanced nutrient concentration and hormonal effects. The positive correlation between these parameters and the uptake of total N, P, K, and S further supports our findings (Table 8).

**Table 8:** Correlation coefficient matrix.

	LN	BN	LP	BP	LK	BK	LS	BS	TDM	TNU	TPU	TKU	TSU	NL60	PH60	Yield	LAI60
NL	1																
NB	0.69***	1															
PL	−0.40**	−0.69***	1														
PB	0.56***	0.59***	−0.16	1													
KL	0.38**	0.73***	−0.85***	0.37**	1												
KB	0.54***	0.86***	−0.81***	0.50***	0.88***	1											
SL	−0.32*	−0.67***	0.88***	−0.31*	−0.90***	−0.85***	1										
SB	0.63***	0.40**	−0.13	0.21	0.06	0.25	−0.05	1									
TDM	−0.09	−0.43**	0.75***	−0.05	−0.77***	−0.67***	0.81***	0.18	1								
TNU	0.59***	0.60***	−0.01	0.55***	0.05	0.26	0.05	0.54***	0.45***	1							
TPU	0.08	−0.18	0.68***	0.46***	−0.54***	−0.41**	0.60***	0.15	0.84***	0.57***	1						
TKU	0.57***	0.62***	−0.21	0.63***	0.33*	0.54***	−0.23	0.49***	0.24	0.83***	0.43**	1					
TSU	0.06	−0.33*	0.68***	−0.04	−0.72***	−0.59***	0.77***	0.45***	0.95***	0.51***	0.77***	0.28*	1				
NL60	−0.01	−0.18	0.42**	0.03	−0.45***	−0.33*	0.52***	0.22	0.57***	0.33*	0.52***	0.19	0.57***	1			
PH60	0.49***	0.30*	0.16	0.43**	−0.11	0.03	0.23	0.49***	0.48***	0.71***	0.56***	0.55***	0.54***	0.40**	1		
Yield	0.25	−0.13	0.59***	0.31*	−0.57***	−0.42**	0.65***	0.33*	0.88***	0.63***	0.90***	0.42**	0.87***	0.56***	0.64***	1	
LAI60	0.53**	0.62***	0.28	0.79***	−0.1	0.33	0.29	0.35	0.74***	0.73***	0.91***	0.82***	0.62***	0.50**	0.74***	0.85***	1

LN, Leaf nitrogen (N) concentration; BN, Bulb N concentration; LP, Leaf phosphorus (P) concentration; BP, Bulb P concentration; LK, Leaf potassium (K) concentration; BK, Bulb K concentration; LS, Leaf sulfur (S) concentration; BS, Bulb S concentration; TDM, total dry matter yield; TNU, Total N uptake; TPU, Total P uptake; TKU, Total K uptake; TSU, Total S uptake; NL60, Number of leaves at 60 Days after transplanting (DAT); PH60, Plant height at 60 DAT; LAI, Leaf area index at 60 DAT. *Significant at *p* = 0.05, **significant at *p* = 0.01, and ***significant at *p* < 0.0001.

The mycelia of the endophytic fungus *S. indica* possibly extend onion roots, thereby facilitating root growth, nutrient absorption, stress tolerance, and systemic resistance (Gill et al., 2016). This symbiotic relationship may have increased the root system’s surface area, thereby enhancing water and nutrient uptake, including N, P, K, and S (Li et al., 2023) and micronutrients (Padash et al., 2016). The increased levels of N, P, K, and S observed in plots receiving 100% RDF combined with *S. indica* inoculation would have boosted overall photosynthetic activity. This, in turn, led to increased biomass accumulation and onion yield. Gill et al. (2016) also documented that the mechanism contributing to increased plant growth and biomass accumulation could be attributed to the enhanced root growth facilitated by the colonized fungi, thereby promoting nutrient absorption from the root zone. Its colonization not only promoted root growth but also increased nutrient absorption in various crops such as sunflower, rapeseed, and rice (Li et al., 2023). Its colonization has been observed to positively impact the growth and biomass of various crop plants, as evidenced by previous studies on *Allium cepa*, *Oryza sativa*, *Saccharum officinarum*, *Abrus precatorius*, *Zea mays*, *Phaseolus vulgaris*, and *Tridax procumbans* (Varma et al., 2012, 2014; Gill et al., 2016; Roylawar et al., 2023).

Meanwhile, PSB have the ability to convert both organic and inorganic P into soluble forms, thereby enhancing its availability to plants (Kalayu, 2019; Rawat et al., 2021) by secreting extracellular enzymes, mineralizing substrates, and producing mineral-dissolving complexes or compounds (Sharma et al., 2013; Silva et al., 2023). Additionally, PSB activity in the rhizosphere may have influenced the production and availability of plant hormones like auxins, cytokinins, and gibberellins (Kenneth et al., 2019; Raza et al., 2019). These hormones are pivotal in governing plant growth and development, including processes such as cell elongation, leaf expansion, and shoot growth. Through the modulation of hormone levels, phosphate-solubilizing bacteria may have indirectly promoted plant height, leaf area expansion, and biomass accumulation (Barazani et al., 2007; Malik et al., 2019). Mangaraj et al. (2022) also documented that increased nutrient availability led to increased conversion of carbohydrates to protein, subsequently enhancing meristematic cellular activity, including cell division and elongation, which manifested morphologically as increased plant growth and ultimately resulted in higher dry matter accumulation and crop yield.

However, inoculation with *S. indica* or PSB in treatments where only 50% RDF was applied did not result in a significant increase in leaf area or plant growth. This suggested that the reduced fertilizer doses may have contributed to a smaller leaf area due to inadequate nutrient availability and uptake, thus limiting the plant’s capacity to capture sunlight and perform photosynthesis effectively (Zhang et al., 2021; Rawat et al., 2022), which has ultimately limited biomass accumulation (Tan et al., 2005). The reduction in biomass production and nutrient uptake may have led to a decrease in bulb yield in plots that received 50% RDF, with or without *S. indica* or PSB inoculation, compared to plots that received 100% RDF, regardless of *S. indica* or PSB inoculation.

The root system was also evaluated for colonization by VAM fungi; however, VAM colonization was not observed in onion roots. The application of mineral fertilizers could have affected the efficacy of VAM fungi in onions. The soils used in this experiment had high concentrations of P, K, S, copper, manganese, and adequate levels of zinc, iron, and boron. The presence of high nutrient levels in the soil may have inhibited the colonization of VAM fungi in onions. Many researchers have documented reduced colonization of arbuscular mycorrhizal fungi in plots treated with mineral fertilizer (Trejo et al., 2021; Fall et al., 2023). However, further investigation is required to understand the reasons for the poor or non-infection of onion roots by VAM fungi, as they are known to be one of the best sources for improving plant growth, development, and crop yield not only in perennial trees but also in various field and vegetable crops.

Additionally, onion yield exhibited significant variation from year to year, with a lower yield recorded in 2018–2019 compared to 2019–2020. The reduced yield in the first year could potentially be attributed to temperature increases at maturity during the harvesting stage (Parthasarathi et al., 2022). The plant’s allocation of energy toward enhancing its antioxidant enzyme system, as part of its defense mechanism against temperature stress, could have potentially contributed to the reduction in onion crop yield. These findings are consistent with the conclusions drawn by Warland et al. (2006) in Brassicaceae, Shamloo et al. (2017) in wheat, and Thangasamy et al. (2021) in garlic, which suggest a notable decline in the yield of cool-season crops in response to even slight temperature increases.

## Conclusion

The comprehensive findings suggest that the application of 100% RDF additionally inoculated with *S. indica* or PSB significantly enhanced plant growth, nutrient uptake, and onion yield compared to both the control and treatments with 50% RDF, with or without microbial inoculation. Conducting field trials across diverse regions and soil types could further validate the efficacy of microbial inoculation and fertilizer dosages under varying environmental conditions and provide more reliable data for practical adoption by farmers. However, it’s noteworthy that VAM did not exhibit satisfactory performance in onion cultivation, as observed in other crops. Additional investigation is necessary to understand the underlying reasons for the poor and inadequate plant colonization or non-infection of onion roots by VAM fungi.

## Data availability statement

The original contributions presented in the study are included in the article/Supplementary material, further inquiries can be directed to the corresponding author.

## Author contributions

TA: Conceptualization, Data curation, Formal analysis, Funding acquisition, Investigation, Methodology, Project administration, Resources, Supervision, Validation, Writing – original draft, Writing – review & editing. KG: Formal analysis, Investigation, Methodology, Writing – original draft. PM: Formal analysis, Investigation, Methodology, Writing – original draft. PS: Data curation, Formal analysis, Investigation, Methodology, Writing – original draft, Writing – review & editing. VG: Conceptualization, Formal analysis, Investigation, Methodology, Resources, Writing – original draft, Writing – review & editing. SG: Investigation, Methodology, Resources, Writing – original draft. VM: Funding acquisition, Project administration, Resources, Writing – original draft.
